# Supplementary material for: Modulation of FLT3-ITD Localization and Targeting of Distinct Downstream Signaling Pathways as Potential Strategies to Overcome FLT3-Inhibitor Resistance
Source: Cells. 2021 Nov 3;10(11):2992. doi: 10.3390/cells10112992 (PMC8616352; doi:10.3390/cells10112992)
Supplement: Supplementary file 1 [file cells-10-02992-s001.zip › cells-1386475-supplementary.pdf]

Supplementary material

Table S1. Detailed statistical comparison of MTS and Annexin V assay in Figure 1: Illustration of apoptosis rates and *p* values in addition to in Figure 1 demonstrated MTS and Annexin V assays for Ba/F3 ITD variants with or without pre-treatment of 1µg/ml tunicamycin for 24 h prior to adding indicated concentrations of 17AAG. IC_{50} for 17-AAG was determined using nonlinear regression model in GraphPad. Two-tailed, unpaired *t*-test was performed for indicated DMSO (0.1% v/v) and tunicamycin conditions (mean values of triplicates obtained from MTS assay). .

Ba/F3-ITD variant, 17AAG in nM	Annexin V negative (DMSO vs. tunicamycin)	P values of t-test (DMSO vs. tunicamycin)	IC_{50} of 17AAG in nM (MTS)
**598/599 (JM, 12)**			
0nM	95.9 vs. 92.2	0.0477	
125nM	94.5 vs. 88.6	0.0271	538.5 vs. 287.3
250nM	91.4 vs. 75.4	0.0047	
500nM	82 vs. 52.1	0.0033	
1000nM	62.7 vs. 38.9	0.0023	
**G613E (TKD1, 33)**			
0nM	95.3 vs. 71.7	0.0006	
125nM	90.9 vs. 41.1	0.0011	172.9 vs. 36.8
250nM	82.8 vs. 32.8	0.0006	
500nM	78.9 vs. 26.6	0.0007	
1000nM	72.7 vs. 24.7	0.0004	
**FV605YF (JM, 14)**			
0nM	95.9 vs. 90.8	0.0029	
125nM	91.7 vs. 74.3	0.0195	279.7 vs. 94.9
250nM	85.6 vs. 52.5	0.0025	
500nM	73.9 vs. 37.6	0.0021	
1000nM	67.6 vs. 33	0.0010	
**E611V (TKD1, 32)**			
0nM	94.6 vs. 92.2	0.1609	
125nM	93.6 vs. 85.1	0.0612	430.6 vs. 234.1
250nM	90.2 vs. 71.3	0.0282	
500nM	81.3 vs. 51.7	0.0137	
1000nM	69.0 vs. 42.8	0.0089	

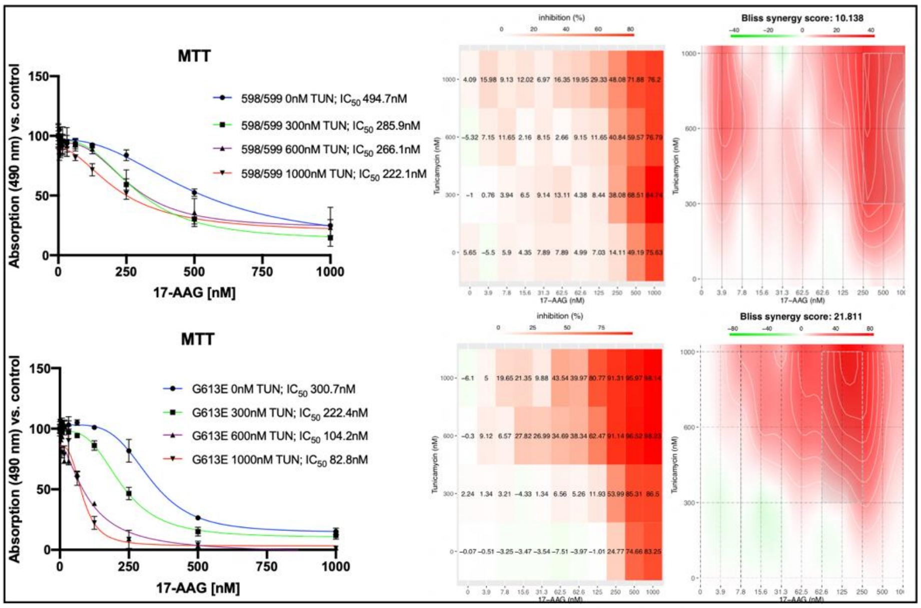

Figure S1: Data of MTS assays (*left*) are demonstrated as relative absorption compared to untreated control. 17-AAG concentration-dependent metabolic activity of both Ba/F3-ITD variants after 24 h pre-incubation with indicated tunicamycin concentrations are shown**.** 17-AAG was added for further 24 h. IC_{50} values are indicated for 17-AAG, dependent on different concentrations of pre-treatment with tunicamycin. Calculation of potential synergy in the right section shows a Bliss synergy score of 10.1 and 21.8 for Ba/F3 598/599 and G613E variant, respectively.

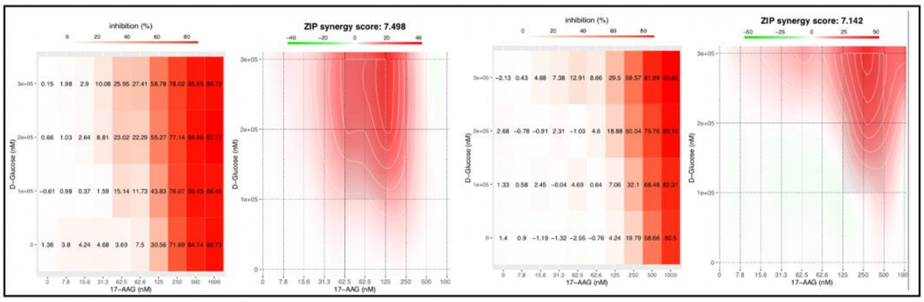

Figure S2: Corresponding calculation of potential synergy effects for MTS assays demonstrated in Figure 2C for Ba/F3 598/599 and G613E variant after 8 h pre-incubation with indicated 2-deoxy-D-glucose concentrations are shown. ZIP synergy score was 7.4 and 7.1 for Ba/F3 G613E and 598/599, respectively.

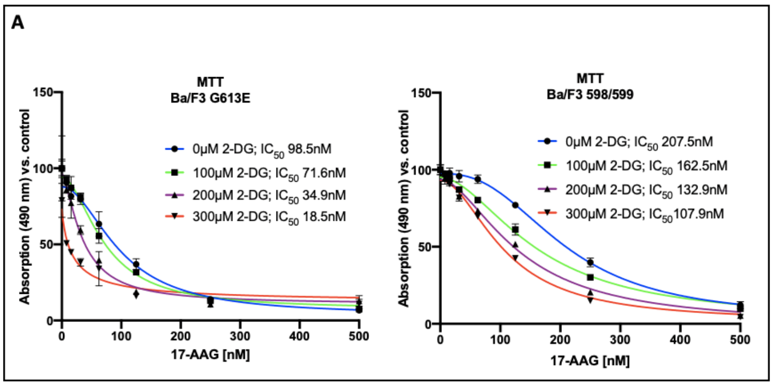

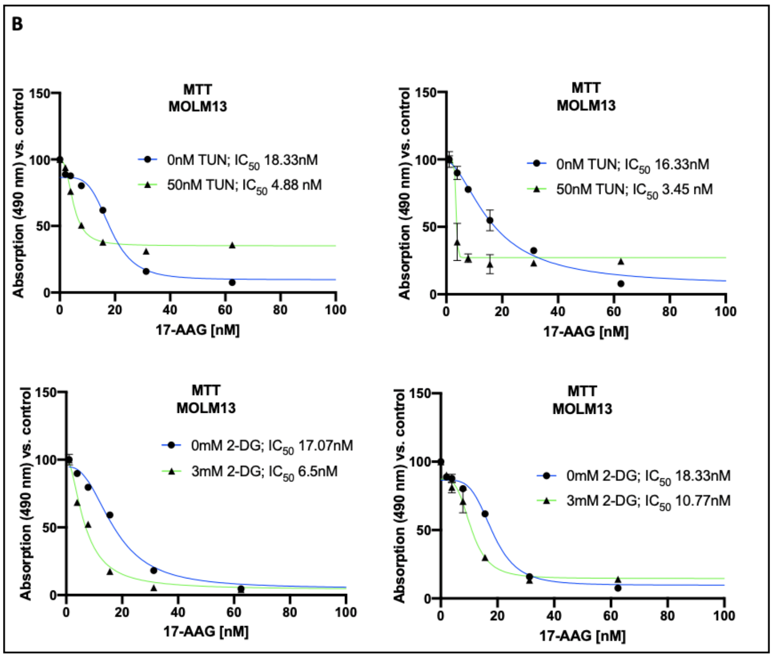

Figure S3: ***A Biological replicates for MTS experiments shown in Figure 2C.*** Data is demonstrated as relative absorption compared to untreated control. 17-AAG concentration-dependent metabolic activity of both Ba/F3-ITD variants after 8 h pre-incubation with indicated 2-deoxy-D-glucose concentrations are demonstrated**.** 17-AAG was added for further 24 h, IC_{50} values are indicated for 17-AAG dependent on different concentrations of pre-treatment with 2-deoxy-D-glucose. Each assay was performed in technical triplicates.

S4: B Biological replicates for MTS experiments shown in Figure 3. Data is demonstrated as relative absorption compared to untreated control. 17-AAG concentration-dependent metabolic activity of MOLM13 cells after 24 h pre-incubation with indicated 2-deoxy-D-glucose and tunicamycin concentrations are demonstrated**.** 17-AAG was added for further 24 h and IC_{50} values are indicated for 17-AAG dependent on different concentrations of pre-treatment with both substances. Each assay was performed in technical triplicates.
